# Remission of Gilles de la Tourette Syndrome after Heat-Induced Dehydration

**DOI:** 10.4172/2329-9096.1000472

**Published:** 2018-06-13

**Authors:** James Robert Brašić, Zoltan Mari, Alicja Lerner, Vanessa Raymont, Eram Zaidi, Dean F. Wong

**Affiliations:** 1 Division of Nuclear Medicine and Molecular Imaging, The Russell H. Morgan Department of Radiology and Radiological Science, Section of High Resolution Brain Positron Emission Tomography Imaging, Johns Hopkins Outpatient Center, Johns Hopkins University School of Medicine, Baltimore, Maryland, USA; 2 Department of Neurology, Johns Hopkins University School of Medicine, Johns Hopkins Hospital, 1800 Orleans Street, Baltimore, Maryland, USA; 3 Center for Drug Evaluation and Research, Food and Drug Administration, 10903 New Hampshire Avenue, Silver Spring, Maryland, USA

**Keywords:** Basal ganglia, Dopamine, Hypothalamus, Substantia nigra, Thermoregulation, Thermostat, Tics

## Abstract

Heat has been reported to exert variable effects on people with Gilles de la Tourette syndrome (TS). At age 24 years, a 32-year-old right-handed man with TS experienced a marked reduction in tics for two years after undergoing dehydration by entering a hot tub at 103°F (39.4°C) to 104°F (40.0°C) for 3 to 4 hours. On the Yale Global Tic Severity Scale (YGTSS) he scored 55 seven months before dehydration and 13 one month after dehydration. An intense heat exposure and dehydration led to an apparent remission in tics. The remission continued without the use of prescribed or nonprescribed medications or substances for two years until tics returned in the worst ever exacerbation after a tetanus immunization. The heat exposure may have altered at least temporarily his thermostat for normal heat-loss mechanisms through dopaminergic pathways from the anterior hypothalamus to the basal ganglia and the substantia nigra. Whether or not that mechanism or some other mechanism relevant to the heat exposure and/or dehydration is at play, the sudden and marked improvement in his tics needs further attention. Prospective testing of the heat and dehydration effect on tics should be pursued.

## Introduction

Anecdotal reports of Gilles de la Tourette syndrome (TS) have suggested either improvement or worsening with heat [1]. Gilles de la Tourette himself reported that acute fever alleviated tics [1,2]. On the other hand, five of ten adult men with TS demonstrated prominent worsening of tics when the ambient temperature was increased from 22°C (71.6°F) to 35°C (95.0°F) [3]. Similarly, some people with autism, another developmental disability, demonstrate improvement of stereotypies and other movements with high temperature [4]. Reports on heat’s effect on tics and stereotypies vary between improvement [1,2] and worsening [3] without reaching a clear consensus.

People with TS typically experience a decrement in tics with dopamine-receptor blocking drugs and an increment in tics from stimulants [1,3]. Release of dopamine in the preoptic area and the anterior hypothalamus plays a role in thermoregulation [3]. Although circuits for heat regulation may not overlap the circuits generating tics in TS, there may be indirect relationships between these circuits. Thus, the release of dopamine may play a role in mediating the effects of heat on tic intensity in people with TS [3].

There have been mixed reports on the rate of spontaneous remission in TS, as defined by greater than 10-point improvement the total score for tics (0-50) including both motor tics (0-25) and phonic tics (0-25) on the Yale Global Tic Severity Scale (YGTSS) [5]. Some investigators found a remission rate as low as 2/31 [6], while others reported somewhat higher rates [7-11]. When observed, spontaneous remission tends to occur at ages younger than the twenties, in patients who had shorter duration of illness, and with overall lower severity scores [12]. Based on the available literature on spontaneous remission, our patient, with moderately severe, long-standing TS in his mid-twenties, represents an exceedingly low probability for spontaneous remission.

## Case Report

A young man participated in multiple assessments during research studies approved by the Institutional Review Board of the Johns Hopkins University School of Medicine, Baltimore, Maryland. He reported that he had induced dehydration several days after the episode without the consent or approval of the study physicians.

### Clinical history

Since the age of 7 years, he experienced motor tics, including eye blinking, grimacing, and movements of head, shoulders, and fingers, and phonic tics, including grunting, snorting, and sniffing. Tics were exacerbated by cold, fatigue, and anxiety. Tics waxed and waned. Since childhood he experienced multiple tics simultaneously such as turning head to his shoulder along with a shoulder shrug.

Since the age of 10 years, he experienced recurrent episodes of 2 to 3 months of daily emesis occurring intermittently throughout the year alternating with complete resolution of the emesis for 2 to 3 months. Each episode of emesis was preceded by an uncontrollable sensation in his throat, a short premonitory awareness of a feeling that the food is going to regurgitate. The emesis occurred only when eating solid foods. Emesis fluid never contained bile or stomach contents. He experienced a sore throat when he had emesis. Emesis occurred after one or two bites of food or midway through each meal. He successfully resumed eating after completing the emesis. Antacids did not relieve the emesis. He undertook diets with foods of various textures without beneficial effects. The symptoms occurred only during eating. He had no other swallowing difficulty, no nausea, no abdominal pain, and no odynophagia.

He was unable to carry parcels with bottles for fear that they could be damaged if he experienced tics.

At age 22, trials of clonidine and topiramate produced minimal effect on tics.

At age 23, he began to occasionally smoke marijuana with relief of tics for 3 to 4 days.

At age 23 years, 5 months, he had his baseline evaluation. For the baseline evaluation please refer to Table 1 for objective assessments.

### Family history

His mother has TS. His sister has obsessive-compulsive disorder. He is married with one child. At age 3 and a half years, his 5-year-old son began to display motor tics, including eye blinks and simultaneous head movements to the shoulder along with shoulder shrugs, and a phonic tic, exaggerated coughing, similar to the proband.

### Other histories

His past medical, surgical, social, developmental, and family histories and his review of symptoms were otherwise noncontributory.

### Clinical course

At the age of 24 years and one month, he deliberately induced dehydration by entering a hot tub of 103°F for 3 and a half hour. During the last one and a half hour in the tub, he noticed that his tics had subsided. He reported that his tics had not recurred. After leaving the hot tub, he vomited clear fluid. He drank water because he was thirsty. He then regurgitated the fluid. After leaving the tub he experienced cramps in his legs, arms, and neck. For two days after leaving the hot tub, his urine was pinkish orange. He felt weak for a couple of days after leaving the hot tub. A month after the dehydration, he scored 13 for motor tics, 0 for phonic tics, and 0 for impairment on the YGTSS [5].

Please refer to Table 1 for a record of the multiple assessments to document his improvement. He was able to work full time as a computer technician. He remained almost completely free of tics for 2 years after heat-induced dehydration. During this remission he denied the use of medications and substances without a prescription. Please refer to supplementary materials for video segments before and after his dehydration.

At the age of 27, he experienced the worst ever acute exacerbation of all tics for two weeks after a tetanus immunization. The tics gradually returned to a mild level. He did not use medications or cannabis.

At the age of 29, he began using Δ^9^-tetrahydrocannabinol (THC) as an inhaled vaper nightly. His tics remain at a mild level. He has emesis approximately every six months. He has no exacerbation of tics with cold. He works ten hour a day for two jobs. He readily carries stock including eggs to shelves for a job without problems.

## Discussion

At age 25, a young man with TS experienced an almost full remission of symptoms sustained for 18 months after undergoing a presumptive dehydration in a hot tub at 103°F (39.4°C) to 104°F (40°C) for 3 to 4 hours.

Cerebral hyperthermia results in an increased blood flow to the brain [33]. Heat exhaustion is a moderate illness resulting from depletion of water and salt associated with core body temperatures between 37°C and 40°C [34]. Heat produced alterations in both excitatory neurotransmitters, aspartate and glutamate, and inhibitory neurotransmitters, gamma-amino-butyric acid (GABA) and glycine [35]. Since dysfunction of the GABA-ergic system is a major effect of heat on the nervous system, the reduced inhibition resulting from damage to GABA neurotransmission may result in increased excitatory neurotransmission in response to heat [35].

Increased excitatory neurotransmission may stimulate further dopaminergic neurotransmission and may predispose a person to develop seizures [35]. Sustained excitatory neurotransmission may have produced a marked phasic increase in dopamine in our patient to result in a lasting effect on thermoregulation by the dopaminergic pathways from the anterior hypothalamus to the basal ganglia and the substantia nigra. Heat and/or dehydration may have led to tiny infarcts in our patient’s dopaminergic pathways from the anterior hypothalamus to the basal ganglia and the substantia nigra. The hyperthermia experienced by this man may have reset his thermostat for normal heat-loss mechanisms through dopaminergic pathways from the anterior hypothalamus to the basal ganglia and the substantia nigra. Whether or not that mechanism or some other mechanism relevant to the heat exposure and/or dehydration is at play, the sudden and marked improvement in his tics needs further attention. Prospective testing of the heat and dehydration effect on tics should be pursued. Clinical trials of dehydration and heat on TS may prove beneficial. The patient has been informed that unsupervised self-induced dehydration may result in serious morbidity and mortality; he has not repeated dehydration.

The patient has noted beneficial effects of tetrahydrocannabinol (THC). He daily utilizes an inhaled vapor of THC nightly. THC, an antagonist of the cannabinoid receptor, has been utilized as a treatment for a spectrum of neuropsychiatric disorders [36]. While acute administration of THC produces increased dopamine release, chronic use reduces dopaminergic neurotransmission [37]. The interactions of THC with the dopaminergic system are complex, and not completely elucidated, however few modes of interactions described result in decreased activity of the dopaminergic system such as: 1) in humans chronic cannabis intake reduces dopamine synthesis capacity in the striatum as seen in a positron emission tomography (PET) study with 18-F-DOPA [38], 2) dopamine release is blunted in chronic human cannabis users [39], and 3) dopamine transporter (DAT) density is reduced in chronic cannabis users as seen in the PET study with 11-C PE2I, a selective DAT radioligand [40].

Because it was postulated that hypersensitivity of dopamine receptors and/or hyperactivity of dopaminergic neurons underlay the pathophysiology of Tourette syndrome [41,42], decrease of dopaminergic tone due to THC effects might have beneficial effects on tics severity. A randomized, double-blind, placebo-controlled crossover single-dose trial of THC in 12 patients with TS demonstrated significant improvements of tics and obsessive-compulsive behaviors [43]. A randomized, double-blind, placebo-controlled clinical trial of THC in 24 patients with TS demonstrated a significant reduction in tics with THC [44]. Nevertheless, systematic reviews concluded that the evidence is insufficient to support the use of THC and other cannabinoids to treat tics and obsessive-compulsive behaviors in people with TS [45,46]. Clinical trials with of THC for TS incorporating monitoring of dopaminergic and cannabinoid neurotransmission are needed to determine if THC is effective for TS.

This case also illustrates retching and vomiting, rare manifestations of tics in TS [47,48].

## Limitations

A placebo effect cannot be excluded in this open label exposure. Although the patient repeatedly exhibited normal temperatures during his baseline evaluation six months before his remission, we lack measured temperatures after the exposure to the hot bath. Given the relatively common occurrence of spontaneous remission, one could make an argument that association between two relatively common occurrences, heat exposure and remission of TS may be somewhat difficult to establish, given the high likelihood of co-occurrence by simple chance. Although he denied recent use of marijuana at each assessment, he may have experienced an effect of prior use of cannabis and related agents containing tetrahydrocannabinol [49]. Nevertheless, the occurrence of heat exposure and remission of TS may be chance. Spontaneous remissions of TS typically occur in adolescence. This patient is older than the typical adolescent to undergo a remission of TS. This single case report does not establish a causal sequence between heat-induced dehydration and remission of TS.

## Supplementary Material

Video Video 1. Segment 1: A 24-year-old man is asked to stand still for 2 minutes. He exhibits many tics of his eyes, face, neck, and hands.
Video 1. Segment 2: Eighteen months later the 25-year-old man is asked to stand still for 2 minutes. This clip was taken 11 months after he apparently experienced dehydration by entering a hot tub at 103°F (39.4°C)-104°F (40.0°F) for 3 to 4 hours. Adventitious movements are minimal at this time.

## Figures and Tables

**Table 1:** Objective assessments of a 25-year-old man 7 months before and 1 and 11 months after an almost full remission following dehydration in a hot tub at 103°F (39.4°C) to 104°F (40.0°C) for 3 to 4 hours.

Instrument	Range of scores	Six months before remission	One month after remission	Eleven months after remission
Urine drug toxicology for tetrahydrocannabis	Negative or positive	Negative	Positive	Positive
Abnormal Involuntary Movement Scale (AIMS) [13-15]	(0,40)	9	0	1
Clinical Global Impression (CGI) Severity Index (SI) [16]	(0,7)	4 Moderately mentally ill	3 Mildly ill	2 Borderline mentally ill
Clinical Global Impression (CGI) Global Improvement (GI) [16]	(0,7)	4 No change	2 Much improved	1 Very much improved
Clinical Global Impression (CGI) Efficacy Index (EI) [16]	(0,16)	13	5	13
Clinical Global Impression (CGI) Therapeutic effect [16]	Not applicable	Unchanged or worse	Moderate	Unchanged or worse
Clinical Global Impression (CGI) Side effects [16]	Not applicable	None	None	None
Clinical Global Impression (CGI) Attention Deficit Disorder (ADD) [17]	(0,6)	1 Borderline	0	1 Borderline
Clinical Global Impression (CGI) Obsessive-Compulsive Disorder (OCD) [17]	(0,6)	3 Moderate	2	2 Mild
Clinical Global Impression (CGI) Tourette syndrome (TS) [17]	(0,6)	3 Moderate	1	1 Borderline
Clinical Global Improvement (CGI) Rater Global Evaluation (RGE) [18]	(1,7)	4 Unchanged	2 Much improved	1 Very much improved
Hillside Akathisia Scale (HAS) Subjective items [13,19]	(0,8)	0	0	0
Hillside Akathisia Scale (HAS) Objective items [13,19]	(0,12)	6	0	0
Hillside Akathisia Scale (HAS) Clinical Global Impression (CGI) Severity of Akathisia (SA) [13,19]	(0,7)	3 Mildly akathisic	1 Normal, not akathisic	1 Normal, not akathisic
Hillside Akathisia Scale (HAS) Clinical Global Impression (CGI) Global Improvement (GI) [13,19]	(0,7)	4 No change	1 Very much improved	1 Very much improved
Magnetic resonance imaging of the brain [20, Table II, page 346]	Normal or abnormal	Normal	Normal	Normal
Maryland Psychiatric Research Center (MPRC) Brief Psychiatric Rating Scale (BPRS) Anchors [21-23]	(20,140)	26	21	21
Movement Disorders Checklist [14]	(0,23)	14	8	0
Myoclonus versus tic checklist [24]	(−2,6)	6	3	0
National Institutes of Mental Health (NIMH) Obsessive-Compulsive Scale (OCS) [18]	(0,15)	5	2	1
Rating Scale for Acute Drug-Induced Akathisia (RSADIA) Subjective [25]	(0,9)	0	0	0
Rating Scale for Acute Drug-Induced Akathisia (RSADIA) Objective [25]	(0,21)	1	0	0
Rating Scale for Acute Drug-Induced Akathisia (RSADIA) Global Rating [25]	(0,3)	0	0	0
Rating scale for drug-induced akathisia (RSDIA) Subjective [26]	(0,6)	0	0	0
Rating scale for drug-induced akathisia (RSDIA) Objective [26]	(0,3)	1	0	0
Rating scale for drug-induced akathisia (RSDIA) Global Clinical Assessment of Akathisia (GCAA) [26]	(0,5)	1	0	0
Rating Scale for Tardive Dyskinesia (RSTD) Face [27]	(16,96)	26	17	18
Rating Scale for Tardive Dyskinesia (RSTD) Neck and trunk [27]	(8,48)	11	8	8
Rating Scale for Tardive Dyskinesia (RSTD) Extremities (upper) [27]	(8,48)	11	8	8
Rating Scale for Tardive Dyskinesia (RSTD) Extremities (lower) [27]	(8,48)	8	8	8
Rating Scale for Tardive Dyskinesia (RSTD) Entire body [27]	(4,24)	4	4	4
Timed Stereotypies Rating Scale [14,28]	(0,1000)	27	2	1
Tourette Syndrome Diagnostic Confidence Index (TSDCI) [29]	(0,100)	61	Missing	82
Yale-Brown Obsessive-Compulsive Scale (Y-BOCS) [30.^31^]	(0,40)	4	10	4
Obsessive-Compulsive Disorder through the application of the criteria current at the time of the study [32] to the Yale-Brown Obsessive-Compulsive Scale (Y-BOCS) [30,31]^a^	(0,1)	0	1	0
Yale Global Tic Severity Scale (YGTSS) Motor [5]	(0,25)	19	13	12
Yale Global Tic Severity Scale (YGTSS) Phonic [5]	(0,25)	9	0	11
Yale Global Tic Severity Scale (YGTSS) Impairment [5]	(0,50)	27	0	9

a Obsessive-Compulsive Disorder (OCD) is diagnosed [32] if on the Y-BOCS [30,31] a person scores 2, 3, or 4 on any of the following items: 1 time spent on obsessions, or 2 interference from obsessions, or 3 distress of obsessions, or 6 time spent on compulsions, or 7 interference from compulsions, or 8 distress of compulsions
